# Allelic and dosage effects of *NHS* in X-linked cataract and Nance–Horan syndrome: a family study and literature review

**DOI:** 10.1186/s13039-021-00566-x

**Published:** 2021-10-07

**Authors:** Caroline Miller, Benjamin G. Gertsen, Audrey L. Schroeder, Chin-To Fong, M. Anwar Iqbal, Bin Zhang

**Affiliations:** 1 Department of Pathology and Laboratory Medicine, University of Rochester Medical Center, 601 Elmwood Ave, Box 608, Rochester, NY 14642 USA; 2 Division of Medical Genetics, University of Rochester Medical Center, Rochester, NY 14642 USA; 3 Department of Pediatrics, University of Rochester Medical Center, Rochester, NY 14642 USA; 4 Department of Medicine, University of Rochester Medical Center, Rochester, NY 14642 USA; 5 Department of Pathology and Pediatrics, University of Rochester Medical Center, 601 Elmwood Ave, Box 608, Rochester, NY 14642 USA

**Keywords:** Nance–Horan syndrome, X-linked cataract, NHS, X chromosome inactivation (XCI), X-autosome translocation, Gene disruption, Dosage effect, Allelic disorders, FISH, Karyotyping, Array-comparative genomic hybridization (aCGH)

## Abstract

Nance–Horan syndrome (NHS) is a rare X-linked dominant disorder caused by mutation in the *NHS* gene on chromosome Xp22.13. (OMIM 302350). Classic NHS manifested in males is characterized by congenital cataracts, dental anomalies, dysmorphic facial features and occasionally intellectual disability. Females typically have a milder presentation. The majority of reported cases of NHS are the result of nonsense mutations and small deletions. Isolated X-linked congenital cataract is caused by non-recurrent rearrangement-associated aberrant *NHS* transcription. Classic NHS in females associated with gene disruption by balanced X-autosome translocation has been infrequently reported. We present a familial NHS associated with translocation t(X;19) (Xp22.13;q13.1). The proband, a 28-year-old female, presented with intellectual disability, dysmorphic features, short stature, primary amenorrhea, cleft palate, and horseshoe kidney, but no NHS phenotype. A karyotype and chromosome microarray analysis (CMA) revealed partial monosomy Xp/partial trisomy 19q with the breakpoint at Xp22.13 disrupting the *NHS* gene. Family history revealed congenital cataracts and glaucoma in the patient’s mother, and congenital cataracts in maternal half-sister and maternal grandmother. The same balanced translocation t(X;19) was subsequently identified in both the mother and maternal half-sister, and further clinical evaluation of the maternal half-sister made a diagnosis of NHS. This study describes the clinical implication of *NHS* gene disruption due to balanced X-autosome translocations as a unique mechanism causing Nance–Horan syndrome, refines dose effects of *NHS* on disease presentation and phenotype expressivity, and justifies consideration of karyotype and fluorescence in situ hybridization (FISH) analysis for female patients with familial NHS if single-gene analysis of *NHS* is negative.

## Introduction

NHS, also known as cataract-dental syndrome, is characterized by bilateral congenital cataracts, dental anomalies such as screwdriver-shaped teeth and bud molars, and dysmorphic facial features such as anteverted pinnae and broad nose [1–3]. Intellectual disability of varying severity may be seen in approximately one in three patients [4]. NHS is inherited in an X-linked manner with heterozygous female carriers often presenting similar but milder phenotypes than affected males [4, 5]. Female carriers typically display posterior Y-sutural lens opacities often with likely congenital cortical riders, but are not expected to have congenital cataracts [6]. NHS is caused by mutation in the *NHS* gene on chromosome Xp22 [7].The *NHS* gene encompasses ~ 650 kb of genomic DNA, coding for a 1630-amino acid putative protein. The NHS protein localizes to sites of cell–cell contact, acts as a novel regulator of actin remodeling and cell morphology likely by orchestrating actin regulatory protein function in response to signaling events during development [8]. A search of The Human Gene Mutation Database at the Institute of Medical Genetics in Cardiff (http://www.hgmd.cf.ac.uk/) reveals fewer than 50 reported mutations in *NHS*. Missense/nonsense mutations and small deletions comprise the majority of reported mutations, with splicing mutations, small insertions and other mutations reported less frequently. Isolated X-linked cataract with other anomalies is an allelic disorder due to altered *NHS* transcription [6, 9].

However, *NHS* disruption due to X-autosome translocation is a rare phenomenon contributing to the NHS related phenotype. A review of the literature revealed a report of a mother and daughter with NHS phenotype as a result of a balanced t(X;1)(p22.13;q22) and preferential inactivation of the normal X chromosome [10]. Such skewed X chromosome inactivation (XCI) is seen in other X-autosome translocations. Inactivation of the abnormal X chromosome, if occurs, would in turn lead to inactivation of the translocated autosomal DNA, ultimately resulting in functional partial monosomy of the inactivated autosomal gene/genes [11]. Preferential inactivation of the normal X chromosome is selected during development and circumvents silencing of autosomal genes associated with profound cellular effects. However, the opposite does occur when translocation disrupts an essential gene on chromosome X and cells with the normal X inactivated are negatively selected [12]. Molecular characterization of disease-associated translocations is important to identify disease etiology. To characterize autosome-X translocations, XCI patterns can be determined by analyzing methylation, gene expression, histone modification, and replication timing [13–15]. Furthermore, breakpoints of balanced translocations can be mapped by a combination of cytogenomic methods (G-banding, FISH, and optical genome mapping) and sequencing (Sanger and NextGen) [16–18].

In this report, we serendipitously identified X-autosome translocation-mediated disruption of the *NHS* gene by analyzing the genomic imbalance responsible for a proband’s phenotype of intellectual disability, multiple congenital anomalies, and primary amenorrhea. Such gene disruption is further characterized using molecular cytogenetics methods in the female balanced translocation carriers of the family. We conclude that *NHS* disruption along with preferential X-inactivation causes familial NHS, and summarize the relationship between *NHS* dosage and disease presentation and phenotype expressivity. The study and literature review also tell us that chromosome and FISH analysis is important to make a genetic diagnosis for NHS manifested in females or isolated X-linked congenital cataracts cases with no mutations identified in the *NHS* gene.

## Clinical report

**Fig. 1:**
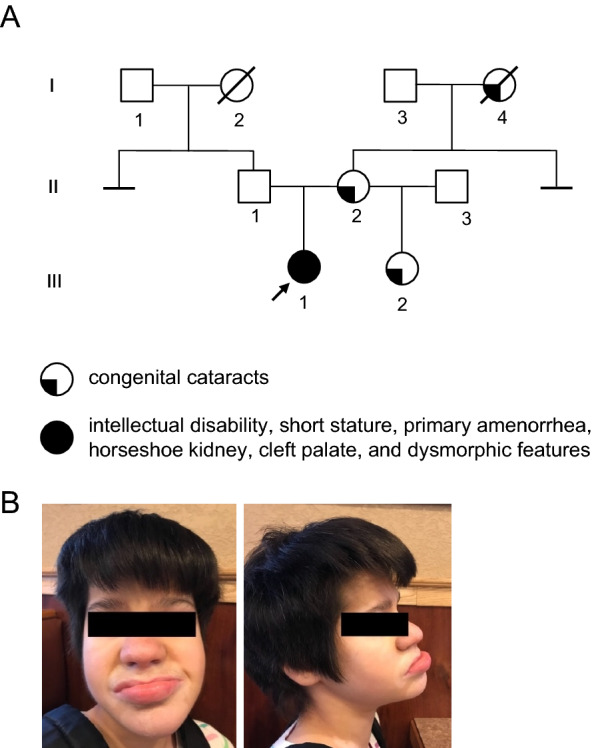
A family affected by familial congenital cataracts. **a** Pedigree. Solid black arrow indicates the proband (filled symbol) with intellectual disability, short stature, primary amenorrhea, horseshoe kidney, cleft palate, and dysmorphic features. Quarter-filled symbols indicates subjects affected by congenital cataracts. **b** Dysmorphic facial features in the proband

The proband (III-1 in Fig. 1a and b) is a 28-year-old female patient with phenotypic characteristics including very short stature (height 121.9 cm), primary amenorrhea, horseshoe kidney, and history of cleft palate repair. The proband was non-verbal and had intellectual disability. Physical examination was notable for dysmorphic features including narrow and short palpebral fissures, thick upper and lower lip, and bitemporal hirsutism, as well as ataxia. There was no history of congenital cataracts, and ophthalmic examination was notable for mild myopia and blepharoptosis. By report, the patient was born full term with birth weight of 0.91 kgs. There was one suspected seizure at age 11 years, history of nephrolithiasis, and history of fundoplication and gastrostomy tube.

A family history was performed and was notable for congenital cataracts and glaucoma in the mother (II-2 in Fig. 1a) and congenital cataracts in the maternal grandmother (I-4 in Fig. 1a) and a half-sister (III-2 in Fig. 1a). Physical examination of the half-sister revealed screwdriver-shaped incisors (Fig. 4a).

## Materials and methods

### G-banding and fluorescence in situ hybridization (FISH)

Peripheral blood samples were cultured using standard cytogenetic methods for 72 h with phytohemagglutinin (PHA) stimulation. Chromosomes were analyzed by G-banding using trypsin digestion and Wright’s staining (GTW). Twenty metaphase spreads were analyzed [17]. The karyotypes were described according to An International System for Human Cytogenomic Nomenclature (ISCN 2016). FISH analyses were performed with standard techniques using SureFISH probes (Agilent Technologies, CA; listed in Fig. 3b and d).

### Array-comparative genomic hybridization (aCGH)

DNA was extracted from the patient’s peripheral blood using QIAamp® DNA Blood Mini Kit (Qiagen, CA). A Nanodrop ND-1000 spectrometer (Thermo Scientific, DE) was used for determination of DNA concentrations. Microarray experiments were performed using the SurePrint G3 Human CGH Microarray 4 × 180 K platform (Agilent Technologies, CA). Data were analyzed and visualized using the Agilent CytoGenomics 4.0 software (Agilent Technologies, CA). Commercially available pooled male DNA (Promega, WI) was used as control DNA. Briefly, after the initial DNA denaturation step, the patient’s DNA and control DNA (500 ng) were labeled with dyes Cyanine-5dUTP and Cyanine-3dUTP, respectively, by using Agilent’s Universal Linkage System (ULS™) technology as per the manufacturer’s recommendations. The hybridization and subsequent steps were performed as per the manufacturer’s recommendations (Agilent Technologies, CA). The slide was scanned in Agilent’s high-resolution Model #G2505C scanner at 3 μm resolution. The scanned image file was directly imported to the Agilent CytogGenomics 4.0 software for the visualization and analysis [17]. All genomic coordinates are based on the Human GRCh37/hg19 Genome Assembly.

### EdU (5-ethynyl-2′-deoxyuridine) incorporation assay for X chromosome inactivation

EdU was incorporated in human lymphocyte culture as described by Luiza Sidelli [15] with modifications as following. The peripheral lymphocytes were cultivated in Chang MF Medium (IrvineScientific, CA) with phytohemagglutinin (PHA) stimulation at 37 °C for 72 h. The cells were treated with 10 μM EdU (Invitrogen, CA) and incubated for additional 2 h at 37 °C. The medium was replaced with a recovery fresh medium, and the cell suspension was incubated for an additional 2 h at 37 °C. The cells were then treated with 10 µg/ml Ethidium Bromide for 1.5 h and 0.09 μg/ml colcemide (IrvineScientific, CA) for 45 min, at 37 °C, followed by 75 mM KCl treatment for 20 min, at 37 °C, and finally fixed in Carnoy fixative (methanol:acetic acid = 3:1). Chromosome spreads, treated with EdU, were dropped onto clean glass slides, and aged on 55 °C hot plate for 20 min, and then stored at room temperature overnight. FISH was performed using the 19q subtel probe (spectrum orange, Abbott Molecular, IL) and the NHS probe (RP11-2K15, spectrum orange, Empire Genomics, NY), according to manufacturer’s protocol. EdU incorporation was detected by the click reaction, in which a copper-catalyzed reaction occurs between the alkyne group of EdU and azide group of Alexa Fluor® 488 dye (Click-iT EdU Alexa Fluor Cell Proliferation Assay kit, Invitrogen, CA). Finally, chromosomes were stained with DAPI. The metaphases were analyzed under a fluorescence microscope (Leica Microsystems, IL) and images were captured using Leica Cytovision Image System (Leica Microsystems, IL).

## Results

**Fig. 2:**
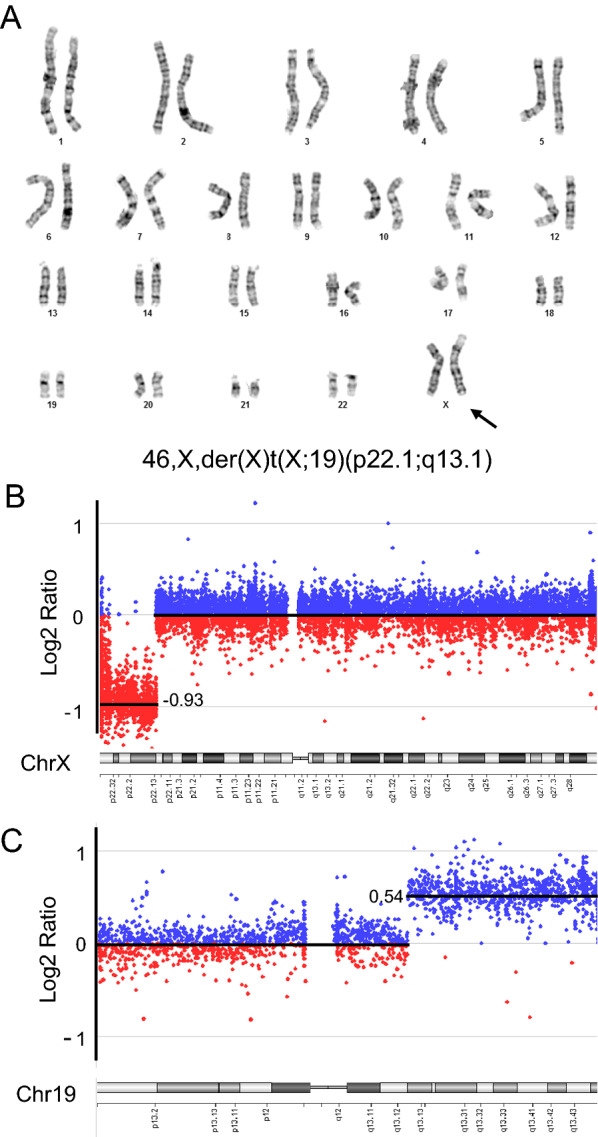
Unbalanced X-autosome translocation causes with intellectual disability, short stature, primary amenorrhea, horseshoe kidney, cleft palate, and dysmorphic features in the proband. **a** Chromosome G-banding analysis identified an unbalanced X-autosome translocation involving chromosome X and chromosome 19 in the proband (III-1). **b** and **c** Chromosome Microarray (CMA) analysis revealed an approximately 17.3 Mb terminal deletion of the short arm of chromosome X (**b**) and an approximately 22.4 Mb terminal gain of the long arm of chromosome 19 (**c**) in the proband. y-axis: Log2 ratio (the log2 ratio of mean fluorescent intensity of proband versus normal control subjects: 0 indicates copy number neutral, − 1 indicates one copy loss, 0.58 indicates one copy gain). x-axis: The band positions of chromosome X and chromosome 19

Chromosome analysis identified in the proband a derivative chromosome X resulted from an unbalanced translocation involving breakpoints on the short arm of chromosome X at band Xp22.1 and the long arm of chromosome 19 at band 19q13.1 (Fig. 2a). aCGH-based chromosome microarray analysis (CMA) revealed a 17.3 Mb partial terminal monosomy Xp (Fig. 2b, 76 OMIM genes) and a 22.4 Mb partial terminal trisomy 19q (Fig. 2c, 599 OMIM genes), consistent with an unbalanced product of translocation t(X;19). The karyotype was defined as 46,X,der(X)t(X;19)(p22.13;q13.1). In this karyotype, the abnormal chromosome X is predicted to be inactivated [19], so the 19q on the der(X)t(X;19) is presumably regulated by the spread of X inactivation. However, such partial autosomal trisomy is not functionally equivalent to disomy as some autosomal genes are resistant to or capable of escaping X inactivation [13]. It is likely that both the terminal Xp deletion and the 19q gain contribute to the proband’s phenotype. For example, the patient had primary amenorrhea which is a characteristic of Turner syndrome, and had cleft palate which can be seen in individuals with partial trisomy 19q. Dosage imbalance of genes on 19q resistant to X-inactivation likely causes additional clinical presentations not typically observed in Turner syndrome, while silencing of other autosomal genes attenuates trisomic phenotypes.

**Fig. 3:**
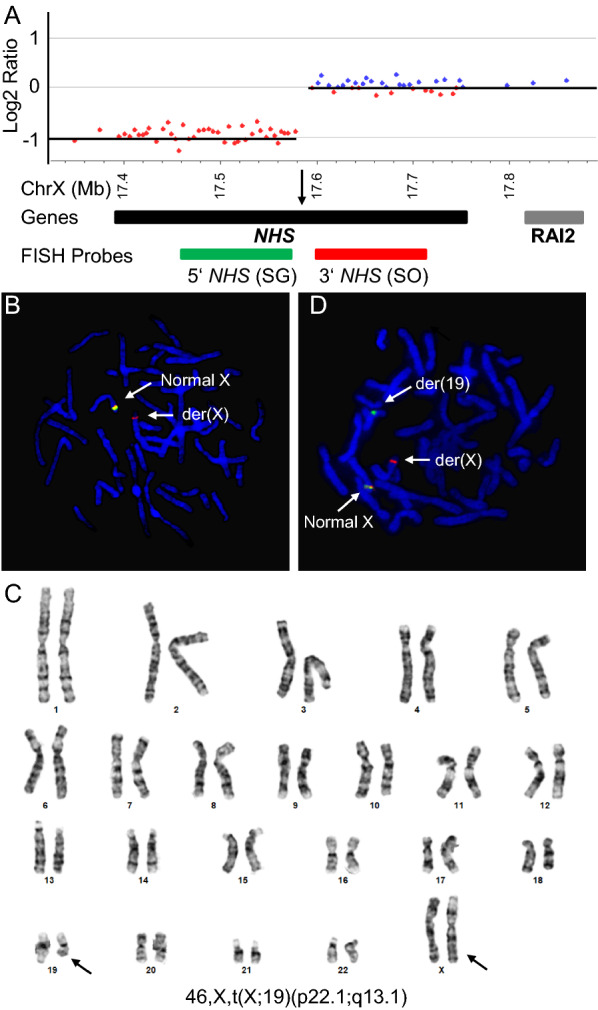
Balanced X-autosome translocation disrupts the *NHS* gene. **a** CMA analysis defines the breakpoint on chromosome X between chrX: 17,579,976–17,597,135, disrupting the *NHS* gene, using the genomic imbalance related to the unbalanced translocation in the proband. The SureFISH *NHS* dual color breakapart probes flanking the breakpoint are indicated (not to scale). 5’ *NHS* SG probe: chrX: 16,968,549–17,396,454; 3’ *NHS* SO probe: chrX: 17,753,424–18,156,264. Arrow indicates the breakpoint. **b** Metaphase FISH analysis shows that in the proband, the normal chromosome X is labeled by both 5’ *NHS* and 3’ *NHS*, while the derivative chromosome X is only labeled by 3’ *NHS*. **c** Chromosome G-banding analysis identified a balanced X-autosome translocation involving chromosome X and chromosome 19 in the maternal half-sister (III-2). **d** Metaphase FISH analysis shows that in III-2, the normal chromosome X is labeled by both 5’ *NHS* and 3’ *NHS*, while the derivative chromosome X is labeled by 3’ *NHS* and the derivative chromosome 19 is labeled by 5’ *NHS*. Blue, DAPI counter-staining; SG, spectrum green; SO, spectrum orange

The family history of congenital cataracts only in females (mother, maternal grandmother, and a maternal half-sister), but not in the proband, suggests that the unbalanced X-autosome translocation is likely inherited maternally. As three affected females in the family do not reportedly present other profound phenotypes, we hypothesize that a balanced reciprocal translocation may cause disruption or dysregulation of a gene (or genes) near the breakpoint causing congenital cataracts. Detailed breakpoint characterization of potential disease-causing balanced translocations has been beneficial to identify and annotate genes important in human development [20]. We therefore examined the CMA data of the proband and identified the breakpoint on chromosome X in the middle of the *NHS* gene (Fig. 3a). Metaphase FISH analysis confirms that 5’ NHS is deleted and 3’ NHS is retained on the derivative chromosome X in the proband (Fig. 3b). We hypothesize that the extreme preferential inactivation of the derivative chromosome X leaves the normal copy on the other chromosome active, therefore does not cause congenital cataract in the proband.

**Fig. 4:**
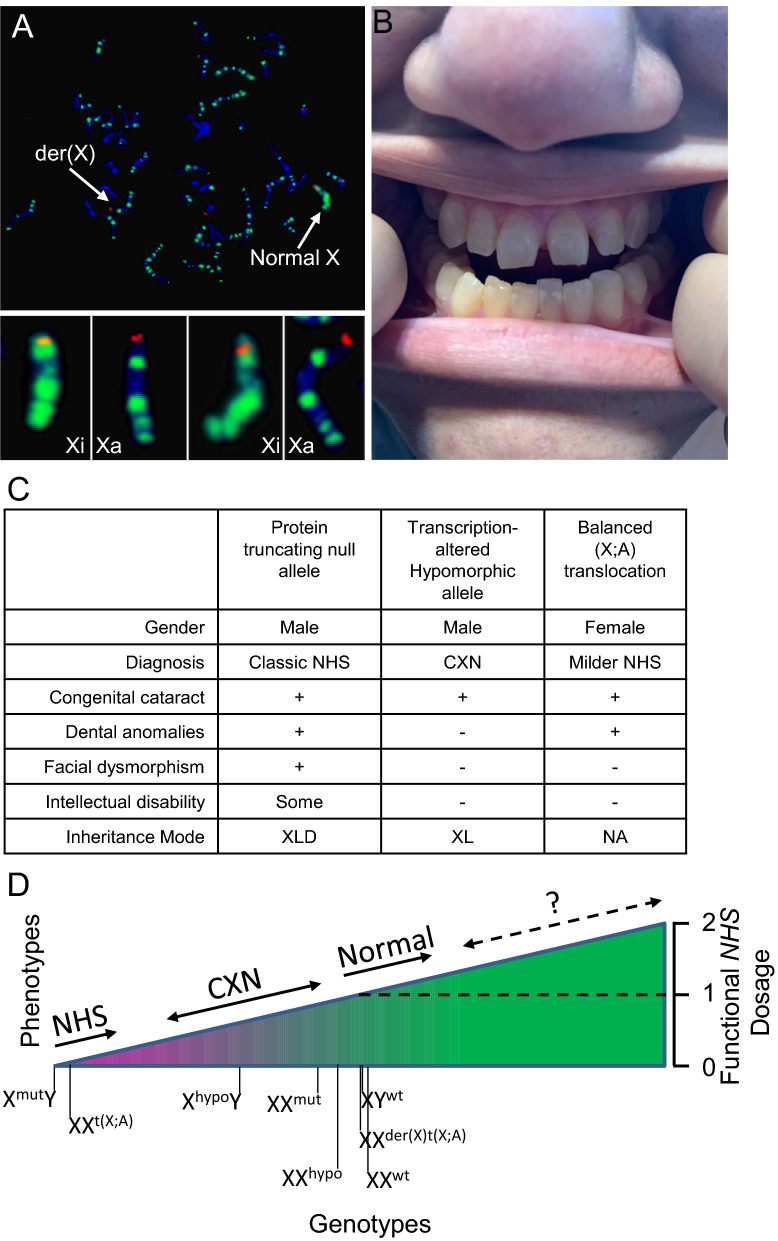
Combination of preferential X inactivation and rearrangement-mediated disruption of *NHS* causes Nance–Horan syndrome in X-autosome balanced translocation carrier females. **a** X-inactivation studies were performed on the maternal half-sister using the incorporation of 5-ethynyl-2′-deoxyuridine (EdU) assay [15]. A skewed X-inactivation pattern with preferential inactivation of the normal X chromosome containing a normal *NHS* gene was revealed. Blue, DAPI counter-staining; Green, EdU; Orange, FISH signals from *NHS* and 19q subtel. Normal X has an *NHS* orange signal located interstitially, while derivative chromosome X has a 19q subtel orange signal located at the tip of the chromosome. Late-replicated chromosomes X were labeled strongly and extensively with EdU green signals across the chromosome relative to the other chromosome X and autosomes. Late replication is associated with X chromosome inactivation. **b** Screwdriver-shaped incisors are seen in the proband’s maternal half-sister (III-2). **c** Phenotypic comparison of allelic disorders caused by different dosages of *NHS*. **d** Quantitative model of dose effects of *NHS* on disease presentation and phenotype expressivity. hypo: *NHS* hypomorphic allele; mut: *NHS* truncating mutation allele; wt: *NHS* wild-type allele. NHS: Nance–Horan synfrome. CXN: X-linked congenital cataract. XLD: X-linked dominant. XL: X-linked. NA: not applicable. + : phenotype present. −: phenotype absent.?: phenotype unknown. XX, female; XY, male

Chromosome and FISH analysis on peripheral blood of the mother and the affected maternal half-sister revealed a balanced translocation t(X;19) (karyotype in Fig. 3c) disrupting one *NHS* allele (metaphase FISH in Fig. 3d) as predicted. X-inactivation analysis using EdU assay [15] showed that the normal copy of chromosome X is preferentially inactivated (Fig. 4a), in contrast to that in the proband. Inactivation of one *NHS* allele on the normal chromosome X and disruption of the other allele on the derivative chromosome X due to the translocation reduce the *NHS* dosage close to zero and cause X-linked cataract in female carriers of this family. Consistently, a follow-up clinical evaluation identified screwdriver-shaped incisors in the maternal half-sister (Fig. 4b), confirming the clinical diagnosis of Nance–Horan syndrome.

## Discussion

We report a familial case with *NHS* disruption due to a translocation t(X;19) (Xp22.13;q13.1). The proband was found to have an unbalanced translocation t(X;19), resulting in partial monosomy Xp/partial trisomy 19q. Phenotypic characteristics in this proband were not consistent with NHS. However, a family history revealed congenital cataracts in three generations of the patient’s maternal lineage, as well as the screwdriver-shaped teeth in the maternal half-sister. These phenotypic findings are typical of NHS [6]. Genetic testing was also provided to the proband’s mother and maternal half-sister, revealing an apparently balanced translocation t(X;19)(Xp22.13;q13.1) in both.

A review of the literature revealed an additional report of *NHS* disruption due to a translocation. In this case, disruption was caused by a balanced (X;1) translocation [21]. As with our report, the patients examined in this study were female. Furthermore, skewed X-inactivation was suggested by lack of *NHS* mRNA expression in one of the patients described. Our case provides additional evidence of a balanced X-autosome translocation with preferential inactivation of normal chromosome X resulting in NHS phenotype in female patients from a single family. The severity of NHS caused by truncating mutations is usually milder in female patients due to its X-linked inheritance pattern [5]. In individuals with unbalanced translocations involving the X chromosome, the abnormal X chromosome is preferentially inactivated [22]. Such non-random X-inactivation prevents functional autosomal monosomy from occurring, but increasing the likelihood of X-linked disorders. Indeed, a preferential inactivation of the normal X chromosome has been reported to cause X-linked disorders in the female carriers of an X-autosome translocation carriers [23–25]. It appears to occur in individuals with a balanced X-autosome translocation disrupting the *NHS* gene, and cause a phenotype consistent with NHS, in particular the facial characteristics and cataracts [21].

In the family of this study, the proband herself had an unbalanced translocation, with partial monosomy Xp and partial trisomy 19q. As a result, the patient’s phenotype reflected aspects of Turner Syndrome (monosomy X) (e.g., primary amenorrhea) [26] and partial trisomy 19 (e.g., cleft palate) [27]. Regarding the female family members, the constellation of congenital cataracts in two carriers of the translocation t(X;19) and one presumptive carrier, and screw-driver shaped teeth in the maternal half-sister, is consistent with a clinical diagnosis of NHS. The *NHS* disruption, identified in the proband and also confirmed as a balanced chromosomal rearrangement in affected family members, suggests this is the familial NHS caused by balanced translocation t(X;19). With no surprise, we found skewed X-inactivation of the normal X chromosome in the proband’s maternal half-sister using the EdU incorporation assay, which provides the molecular mechanism of the NHS manifested in females.

NHS is most commonly due to nonsense mutations and small deletions [21, 28] and only one missense mutation has been reported [29]. *NHS* transcriptional alteration, likely reduction due to complex gene rearrangements and/or intronic enhancer deletions, may cause X-linked cataract, an allelic disorder without other features such as dental anomalies, dysmorphism, and developmental delay [6] (Fig. 4c). Taken altogether, the genetic data suggest that NHS is a dosage-sensitive gene. Complete loss of *NHS* expression causes classic NHS (Fig. 4c and d), while one or slightly more than one functional allele of *NHS* (e.g., XY^wt^ and XX^wt^) is sufficient for normal development as in wild-type XY male or XX female (X-inactivation-mediated gene silencing may not be 100% effective). Individuals with a hypomorphic allele (e.g., X^hypo^Y and XX^mut^) have *NHS* expression lower than normal but not completely absent, will present X-linked cataract (Fig. 4c and d). Individuals with XX^hypo^ with a random X inactivation have a *NHS* dosage more close to normal, so may present very mild phenotypes if any (Fig. 4c and 4d). X-inactivation may not silence gene expression completely, so females with XX^t(X;A)^ disrupting the *NHS* gene may have an ultra-low expression of *NHS* close to that in X^null^Y males and present a phenotype of NHS that could be slightly milder (Fig. 4c and d). A literature search has not identified reports of diseases associated with focal copy number gains encompassing the entirety of the *NHS* gene, suggesting that the developmental consequence of *NHS* functional copy number gain (e.g., two functional copies) remains uncertain (Fig. 4d).

Traditionally, mutations seen in the *NHS* gene have been detected using sequencing [30, 31]. While this assay is useful in detecting single nucleotide variants and small deletions and insertions (indels), it is not effective at identifying aneuploidy, balanced and unbalanced translocations. FISH testing is powerful to identify recurrent balanced translocations involving specific genes or genomic loci. The translocation-mediated *NHS* disruption is rare, but female carriers may present NHS phenotype or X-linked cataract due to non-random X inactivation favoring inactivation of the normal X chromosome. This study demonstrates the importance of karyotype and FISH analysis for patients with NHS or X-linked cataract affecting females. This study also discusses the clinical implication of *NHS* disruption due to balanced X-autosome translocations as a unique mechanism causing Nance–Horan syndrome, and refines dose effects of the *NHS* gene on disease presentation and phenotype expressivity, important for genetic counseling.

## Data Availability

The raw data of CGH and FISH were available upon request.
